# Supplementary material for: TastepepAI: An artificial intelligence platform for taste peptide de novo design
Source: PLoS Comput Biol. 2025 Oct 16;21(10):e1013602. doi: 10.1371/journal.pcbi.1013602 (PMC12543283; doi:10.1371/journal.pcbi.1013602)
Supplement: S1 File — (ZIP) [file pcbi.1013602.s006.zip › Supplementary Data 2/TaPep52 HPLC.doc]

**HPLC REPORT**

| Sample: | NJP93355 TaPep52 SS-6 | | | Analyzed date: | 2024-9-22 |
| --- | --- | --- | --- | --- | --- |
| Analyst: | WGJ | | | Reconstitution: | ACN:H_2_O=3:1 |
| Lot. No.: | P240920-MX1172269 | | | | |
| Column: | 4.6×250mm,Agela C18-5 | | | | |
| Solvent A | A: 0.1% Trifluoroacetic Acid in 100% Acetonitrile | | | | |
| Solvent B | B: 0.1% Trifluoroacetic Acid in 100% Water | | | | |
| Gradient: |  | A | B |  | |
|  | 0.0min | 19% | 81% |  | |
|  | 25.0min | 44% | 56% |  | |
|  | 25.1min | 100% | 0% |  | |
|  | 30.0min | Stop | |  | |
| Volume: | 5μl | | |  | |
| Wavelength: | 220nm | | |  | |
| Flow rate: | 1.0ml/min | | |  | |
|  | | | | | |
| ────────────────────────────────────────────────────  Peak Time Conc Area Height .  ────────────────────────────────────────────────────.  1 10.052 0.861 68208 10654  2 10.967 0.5538 43867 65969  3 11.052 97.49 7722710 727833  4 11.279 1.096 86808 43217  Total 100 7921593 847673 | | | | | |
